# Crystal structure of benzyl 3-oxo-2-oxa-5-aza­bicyclo­[2.2.1]heptane-5-carboxyl­ate

**DOI:** 10.1107/S2056989015010464

**Published:** 2015-06-06

**Authors:** Suvratha Krishnamurthy, Venkataprasad Jalli, Tarun Chand Vagvala, Tetsuji Moriguchi, Akihiko Tsuge

**Affiliations:** a Department of Applied Chemistry, Graduate School of Engineering, Kyushu Institute of Technology, 1-1 sensui-cho tobata-ku kitakyushu 804-8550, Japan; b Department of Biological Functions and Systems Graduate School of Life Science and Systems Engineering, Kyushu Institute of Technology, 2-4 Hibikino, Wakamatsu-ku, Kitakyushu 808-0196, Japan

**Keywords:** crystal structure, 4-hy­droxy­proline, C—H⋯O inter­actions

## Abstract

The title compound, C_13_H_13_NO_4_ (also known as *N*-benzyl­oxycarbonyl-4-hy­droxy-l-proline lactone), crystallizes with two mol­ecules in the asymmetric unit. They have slightly different conformations: the fused ring systems almost overlap, but different C—O—C—C torsion angles for the central chains of −155.5 (2) and −178.6 (2)° lead to different twists for the terminal benzene ring. In the crystal, the mol­ecules are linked by C—H⋯O inter­actions, generating a three-dimensional network. The absolute structure was established based on an unchanging chiral centre in the synthesis.

## Related literature   

For biological background, see: Dickens *et al.* (2008 ▸); Erdmann & Wennemers (2011 ▸); Krishnamurthy *et al.* (2014 ▸); Gómez-Vidal & Silverman (2001 ▸). For the synthesis, see: Lombardo *et al.* (2012 ▸).
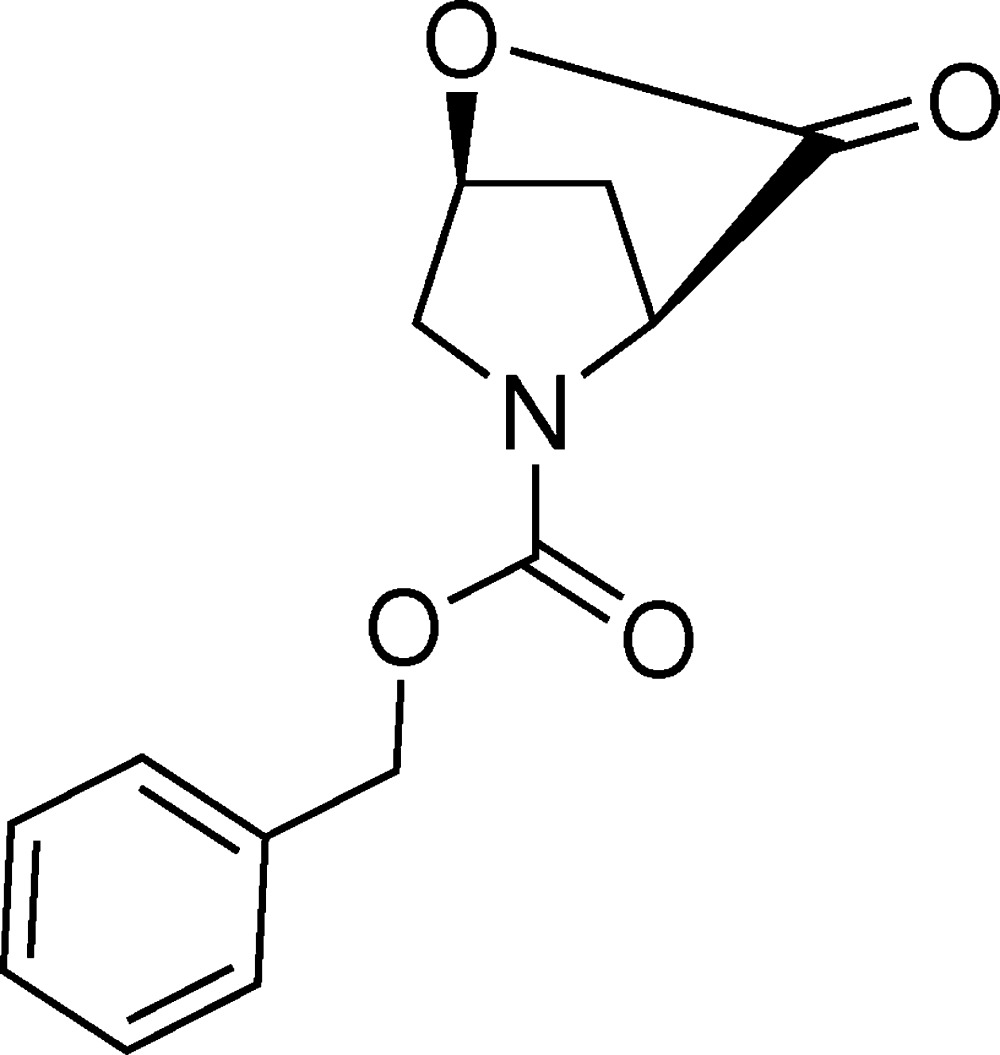


## Experimental   

### Crystal data  

C_13_H_13_NO_4_
*M* *_r_* = 247.24
Monoclinic, 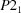

*a* = 11.212 (2) Å
*b* = 8.8943 (16) Å
*c* = 12.258 (2) Å
β = 105.345 (2)°
*V* = 1178.8 (4) Å^3^
*Z* = 4
Mo *K*α radiation
μ = 0.10 mm^−1^
*T* = 90 K
0.40 × 0.35 × 0.30 mm

### Data collection  

Bruker APEX II KY CCD diffractometer
Absorption correction: multi-scan (*SADABS*; Bruker, 2009 ▸) *T*_min_ = 0.709, *T*_max_ = 0.969
11252 measured reflections
4157 independent reflections
4079 reflections with *I* > 2σ(*I*)
*R*_int_ = 0.035

### Refinement  

*R*[*F* ^2^ > 2σ(*F* ^2^)] = 0.027
*wR*(*F* ^2^) = 0.073
*S* = 1.06
4157 reflections
325 parameters
1 restraint
H-atom parameters constrained
Δρ_max_ = 0.19 e Å^−3^
Δρ_min_ = −0.17 e Å^−3^

### 

Data collection: *APEX2* (Bruker, 2009 ▸); cell refinement: *SAINT* (Bruker, 2009 ▸); data reduction: *SAINT*; program(s) used to solve structure: *SHELXS97* (Sheldrick, 2008 ▸); program(s) used to refine structure: *SHELXL97* (Sheldrick, 2008 ▸); molecular graphics: *Mercury* (Macrae *et al.*, 2008 ▸); software used to prepare material for publication: *SHELXL97*.

## Supplementary Material

Crystal structure: contains datablock(s) global, I. DOI: 10.1107/S2056989015010464/hb7435sup1.cif

Structure factors: contains datablock(s) I. DOI: 10.1107/S2056989015010464/hb7435Isup2.hkl

**Fig. 1.**
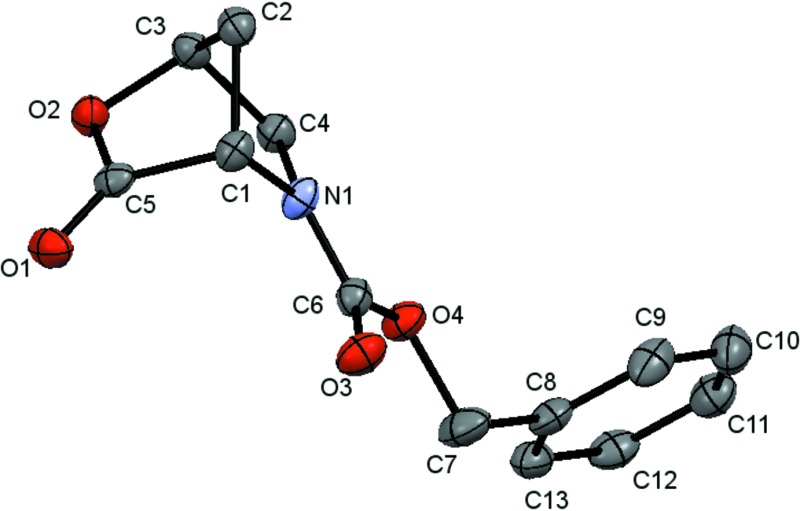
Molecular configuration for the title compound with displacement ellipsoids drawn at the 50% probability level. Hydrogen atoms are omitted for clarity.

. DOI: 10.1107/S2056989015010464/hb7435fig1.tif
Mol­ecular configuration for the title compound with displacement ellipsoids drawn at the 50% probability level. Hydrogen atoms are omitted for clarity.

**Fig. 2.**
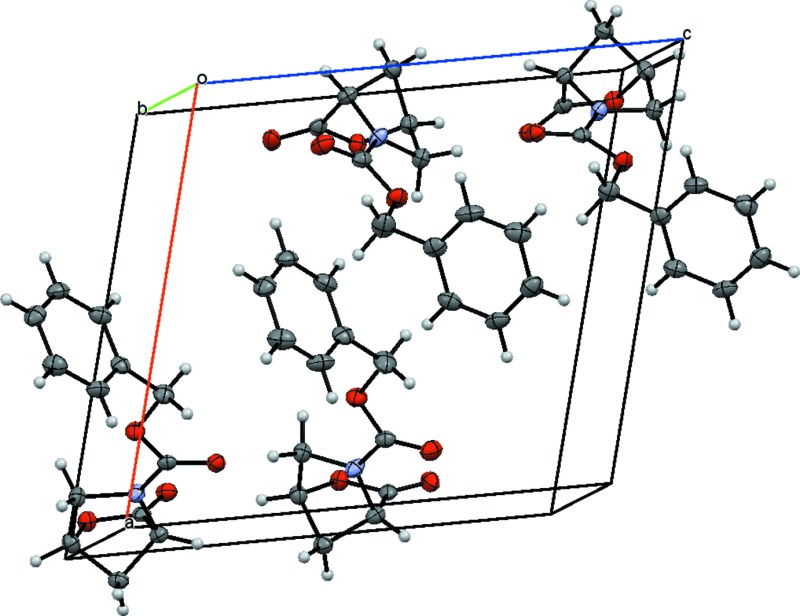
Crystal packing diagram of the title compound.

. DOI: 10.1107/S2056989015010464/hb7435fig2.tif
Crystal packing diagram of the title compound.

**Fig. 3.**
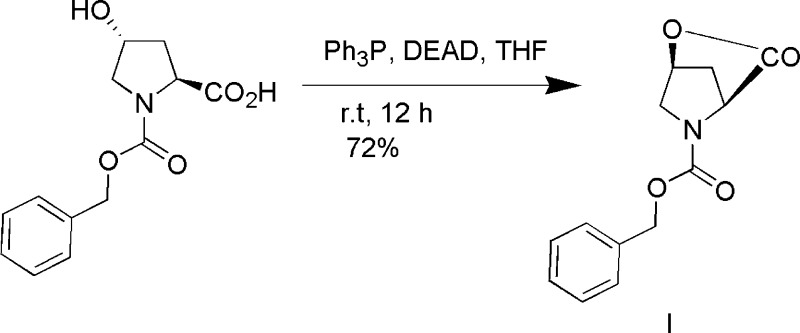
Synthetic scheme for the title compound (I)

. DOI: 10.1107/S2056989015010464/hb7435fig3.tif
Synthetic scheme for the title compound (I)

CCDC reference: 1402251

Additional supporting information:  crystallographic information; 3D view; checkCIF report

## Figures and Tables

**Table 1:** Hydrogen-bond geometry (, )

*D*H*A*	*D*H	H*A*	*D* *A*	*D*H*A*
C1H1O5^i^	0.98	2.42	3.3493(18)	159
C2H2*A*O7^ii^	0.97	2.46	3.2116(18)	134
C3H3O5^iii^	0.98	2.37	3.2816(18)	155
C4H4*B*O7^iii^	0.97	2.39	3.3408(18)	168
C15H15*A*O3^iv^	0.97	2.44	3.1382(19)	128
C16H16O1^v^	0.98	2.49	3.2207(18)	131
C26H26O6^vi^	0.93	2.58	3.4584(19)	157

Symmetry codes: (i) 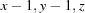; (ii) 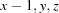; (iii) 
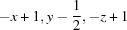
; (iv) 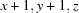; (v) 
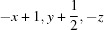
; (vi) 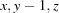.

## supplementary crystallographic information

### S1. Chemical context

Four possible stereoisomers exists for 4-hy­droxy­proline. The (2*S*,4*R*)-isomer is mainly found in collagen and its presence in collagen is a very important factor for its triple helix stabilization (Erdmann & Wennemers, 2011). Moreover (2*S*,4*S)-* of 4-hy­droxy­proline isomer was found to have anti­cancer activity (Dickens *et al.* 2008)

For related derivatives and synthesis see (Krishnamurthy *et al.* 2014, Gomez-Vidal and Silverman, 2001)

### S2. Synthesis and crystallization

(I) was prepared in exactly the same manner as described by (Lombardo *et al.* 2012). To a solution of Z-Hyp-OH (3.0 g, 11.3 mmol) and tri­phenyl­phosphine (3.5 g, 13.3 mmol) in dry THF (50 mL) at 0 °C, was added DEAD (40% in toluene, 5.7 mL, 12.4 mmol), under argon. The resulting solution was warmed to room temperature and stirred. After 12 h, the solvent was evaporated to give a crude residue which was purified directly by flash chromatography on silica gel (EtOAc:hexane, 40:60, v/v) to give I as a white solid ( 2 g, 72 %). (^1^H NMR matched Lombardo *et al.* 2012)

Single crystals were obtained by vapour diffusion method at room temperature, i.e., pentane vapour was allowed to slowly diffuse into a EtOAc (0.3 ml) solution of I at room temperature. Single crystals were obtained after three days.

### S3. Refinement details

Crystal data, data collection and structure refinement details are summarized in Table 1.

### Crystal data

**Table:**

C_13_H_13_NO_4_	*Z* = 4
*M**_r_* = 247.24	*F*(000) = 520
Monoclinic, *P*2_1_	*D*_x_ = 1.393 Mg m^−^^3^
*a* = 11.212 (2) Å	Mo *K*α radiation, λ = 0.71073 Å
*b* = 8.8943 (16) Å	µ = 0.10 mm^−^^1^
*c* = 12.258 (2) Å	*T* = 90 K
β = 105.345 (2)°	Prism, colorless
*V* = 1178.8 (4) Å^3^	0.40 × 0.35 × 0.30 mm

### Data collection

**Table:**

Bruker APEX II KY CCD diffractometer	4157 independent reflections
Radiation source: fine-focus sealed tube	4079 reflections with *I* > 2σ(*I*)
Graphite monochromator	*R*_int_ = 0.035
Detector resolution: 8.333 pixels mm^-1^	θ_max_ = 25.0°, θ_min_ = 1.7°
φandω–scans	*h* = −13→13
Absorption correction: multi-scan (*SADABS*; Bruker, 2009)	*k* = −10→10
*T*_min_ = 0.709, *T*_max_ = 0.969	*l* = −14→14
11252 measured reflections	

### Refinement

**Table:**

Refinement on *F*^2^	Primary atom site location: structure-invariant direct methods
Least-squares matrix: full	Secondary atom site location: difference Fourier map
*R*[*F*^2^ > 2σ(*F*^2^)] = 0.027	Hydrogen site location: inferred from neighbouring sites
*wR*(*F*^2^) = 0.073	H-atom parameters constrained
*S* = 1.06	*w* = 1/[σ^2^(*F*_o_^2^) + (0.0404*P*)^2^ + 0.1656*P*] where *P* = (*F*_o_^2^ + 2*F*_c_^2^)/3
4157 reflections	(Δ/σ)_max_ < 0.001
325 parameters	Δρ_max_ = 0.19 e Å^−^^3^
1 restraint	Δρ_min_ = −0.16 e Å^−^^3^

### Special details

**Table:**

Geometry. All e.s.d.'s (except the e.s.d. in the dihedral angle between two l.s. planes) are estimated using the full covariance matrix. The cell e.s.d.'s are taken into account individually in the estimation of e.s.d.'s in distances, angles and torsion angles; correlations between e.s.d.'s in cell parameters are only used when they are defined by crystal symmetry. An approximate (isotropic) treatment of cell e.s.d.'s is used for estimating e.s.d.'s involving l.s. planes.
Refinement. Refinement of *F*^2^ against ALL reflections. The weighted *R*-factor *wR* and goodness of fit *S* are based on *F*^2^, conventional *R*-factors *R* are based on *F*, with *F* set to zero for negative *F*^2^. The threshold expression of *F*^2^ > σ(*F*^2^) is used only for calculating *R*-factors(gt) *etc*. and is not relevant to the choice of reflections for refinement. *R*-factors based on *F*^2^ are statistically about twice as large as those based on *F*, and *R*- factors based on ALL data will be even larger.

### Fractional atomic coordinates and isotropic or equivalent isotropic displacement parameters (Å^2^)

**Table:**

	*x*	*y*	*z*	*U*_iso_*/*U*_eq_	
C1	0.03159 (12)	0.45789 (16)	0.37070 (12)	0.0215 (3)	
H1	−0.0256	0.4019	0.31	0.026*	
C2	−0.02168 (13)	0.52285 (17)	0.46339 (12)	0.0235 (3)	
H2A	−0.0862	0.5968	0.4356	0.028*	
H2B	−0.0493	0.4465	0.5077	0.028*	
C3	0.10287 (13)	0.59254 (17)	0.52422 (12)	0.0229 (3)	
H3	0.1023	0.6507	0.592	0.027*	
C4	0.19041 (13)	0.45866 (17)	0.54550 (11)	0.0229 (3)	
H4A	0.2754	0.4888	0.5535	0.027*	
H4B	0.1852	0.4018	0.6116	0.027*	
C5	0.09010 (12)	0.59924 (16)	0.33634 (11)	0.0210 (3)	
C6	0.19212 (13)	0.25806 (16)	0.39927 (12)	0.0218 (3)	
C7	0.35825 (16)	0.0823 (2)	0.44690 (14)	0.0356 (4)	
H7A	0.4176	0.1195	0.4083	0.043*	
H7B	0.3012	0.0154	0.3961	0.043*	
C8	0.42360 (14)	0.00050 (16)	0.55255 (13)	0.0265 (3)	
C9	0.35980 (13)	−0.10101 (17)	0.60196 (15)	0.0319 (4)	
H9	0.2765	−0.1189	0.5683	0.038*	
C10	0.41768 (15)	−0.17599 (18)	0.70017 (15)	0.0341 (4)	
H10	0.3731	−0.2425	0.7328	0.041*	
C11	0.54297 (15)	−0.15203 (18)	0.75052 (14)	0.0310 (3)	
H11	0.5828	−0.2035	0.8161	0.037*	
C12	0.60744 (13)	−0.05120 (18)	0.70219 (13)	0.0290 (3)	
H12	0.6911	−0.0349	0.7354	0.035*	
C13	0.54857 (13)	0.02589 (18)	0.60467 (13)	0.0281 (3)	
H13	0.5926	0.095	0.5737	0.034*	
C14	0.96660 (12)	0.95675 (15)	0.18254 (11)	0.0199 (3)	
H14	0.9969	0.9291	0.2625	0.024*	
C15	1.06237 (13)	0.96354 (17)	0.11437 (12)	0.0228 (3)	
H15A	1.1262	1.0383	0.1421	0.027*	
H15B	1.0989	0.8666	0.1065	0.027*	
C16	0.96511 (13)	1.01286 (16)	0.00832 (12)	0.0230 (3)	
H16	0.9969	1.0321	−0.0575	0.028*	
C17	0.86734 (13)	0.88969 (16)	−0.00859 (11)	0.0227 (3)	
H17A	0.7863	0.926	−0.0497	0.027*	
H17B	0.8892	0.8029	−0.0472	0.027*	
C18	0.90739 (12)	1.11153 (16)	0.15699 (11)	0.0200 (3)	
C19	0.81590 (12)	0.74552 (15)	0.15094 (12)	0.0190 (3)	
C20	0.66990 (14)	0.54756 (16)	0.09787 (12)	0.0257 (3)	
H20A	0.6144	0.5876	0.1394	0.031*	
H20B	0.7242	0.4745	0.145	0.031*	
C21	0.59844 (12)	0.47712 (16)	−0.01037 (12)	0.0224 (3)	
C22	0.47838 (13)	0.52208 (17)	−0.06415 (13)	0.0259 (3)	
H22	0.4391	0.5936	−0.0305	0.031*	
C23	0.41709 (13)	0.46052 (18)	−0.16784 (13)	0.0280 (3)	
H23	0.3365	0.49	−0.2028	0.034*	
C24	0.47492 (14)	0.35578 (17)	−0.21940 (13)	0.0293 (3)	
H24	0.4339	0.3158	−0.2894	0.035*	
C25	0.59509 (15)	0.31033 (18)	−0.16600 (14)	0.0310 (3)	
H25	0.6349	0.2404	−0.2006	0.037*	
C26	0.65512 (13)	0.36930 (17)	−0.06153 (13)	0.0262 (3)	
H26	0.7344	0.3364	−0.0251	0.031*	
N1	0.13918 (11)	0.37446 (13)	0.43984 (10)	0.0227 (3)	
N2	0.87336 (11)	0.85642 (14)	0.11057 (9)	0.0213 (2)	
O1	0.10735 (9)	0.63621 (12)	0.24784 (8)	0.0278 (2)	
O2	0.13217 (9)	0.68041 (11)	0.43299 (8)	0.0233 (2)	
O3	0.15344 (9)	0.20350 (13)	0.30604 (9)	0.0302 (2)	
O4	0.29114 (9)	0.20752 (12)	0.47981 (8)	0.0267 (2)	
O5	0.85762 (8)	1.19197 (12)	0.21078 (8)	0.0252 (2)	
O6	0.91040 (9)	1.14515 (11)	0.04972 (8)	0.0232 (2)	
O7	0.82890 (8)	0.71808 (11)	0.25057 (8)	0.0224 (2)	
O8	0.74174 (9)	0.66846 (11)	0.06459 (8)	0.0244 (2)	

### Atomic displacement parameters (Å^2^)

**Table:**

	*U*^11^	*U*^22^	*U*^33^	*U*^12^	*U*^13^	*U*^23^
C1	0.0192 (6)	0.0193 (7)	0.0234 (7)	0.0006 (6)	0.0013 (5)	−0.0015 (6)
C2	0.0206 (7)	0.0251 (7)	0.0246 (7)	0.0012 (6)	0.0055 (6)	0.0036 (6)
C3	0.0257 (7)	0.0239 (7)	0.0195 (7)	0.0011 (6)	0.0068 (6)	0.0003 (6)
C4	0.0246 (7)	0.0232 (7)	0.0200 (7)	0.0005 (6)	0.0043 (5)	0.0000 (6)
C5	0.0185 (6)	0.0229 (7)	0.0204 (7)	0.0071 (5)	0.0031 (5)	0.0010 (5)
C6	0.0188 (6)	0.0196 (7)	0.0268 (7)	−0.0004 (5)	0.0056 (5)	0.0016 (6)
C7	0.0374 (9)	0.0358 (8)	0.0343 (8)	0.0174 (7)	0.0109 (7)	−0.0006 (7)
C8	0.0245 (7)	0.0228 (8)	0.0325 (8)	0.0074 (6)	0.0081 (6)	−0.0035 (6)
C9	0.0171 (7)	0.0253 (8)	0.0502 (10)	−0.0003 (6)	0.0031 (6)	−0.0034 (7)
C10	0.0282 (8)	0.0255 (8)	0.0497 (10)	0.0006 (6)	0.0123 (7)	0.0060 (7)
C11	0.0278 (8)	0.0301 (8)	0.0329 (8)	0.0075 (6)	0.0042 (6)	0.0019 (6)
C12	0.0172 (7)	0.0322 (8)	0.0360 (8)	0.0036 (6)	0.0041 (6)	−0.0069 (7)
C13	0.0242 (7)	0.0253 (7)	0.0383 (9)	0.0007 (6)	0.0144 (7)	−0.0027 (7)
C14	0.0181 (6)	0.0208 (7)	0.0198 (6)	−0.0006 (5)	0.0032 (5)	−0.0009 (5)
C15	0.0201 (7)	0.0227 (7)	0.0262 (7)	0.0004 (6)	0.0073 (5)	−0.0026 (6)
C16	0.0262 (7)	0.0216 (7)	0.0239 (7)	0.0022 (6)	0.0111 (6)	−0.0007 (6)
C17	0.0253 (7)	0.0238 (7)	0.0183 (7)	−0.0008 (6)	0.0047 (5)	−0.0011 (6)
C18	0.0147 (6)	0.0220 (7)	0.0225 (7)	−0.0046 (5)	0.0034 (5)	−0.0025 (5)
C19	0.0149 (6)	0.0178 (7)	0.0238 (7)	0.0030 (5)	0.0043 (5)	−0.0001 (5)
C20	0.0267 (7)	0.0222 (8)	0.0284 (8)	−0.0069 (6)	0.0075 (6)	0.0006 (6)
C21	0.0213 (7)	0.0200 (7)	0.0266 (7)	−0.0057 (6)	0.0077 (5)	0.0017 (6)
C22	0.0224 (7)	0.0222 (7)	0.0345 (8)	0.0009 (6)	0.0103 (6)	0.0006 (6)
C23	0.0174 (7)	0.0278 (7)	0.0362 (8)	−0.0019 (6)	0.0028 (6)	0.0057 (7)
C24	0.0284 (8)	0.0271 (8)	0.0295 (8)	−0.0064 (6)	0.0026 (6)	−0.0028 (6)
C25	0.0298 (8)	0.0269 (8)	0.0376 (8)	0.0013 (6)	0.0112 (6)	−0.0057 (7)
C26	0.0178 (7)	0.0252 (7)	0.0350 (8)	0.0008 (6)	0.0061 (6)	0.0018 (6)
N1	0.0230 (6)	0.0191 (6)	0.0225 (6)	0.0033 (5)	0.0001 (5)	−0.0003 (5)
N2	0.0221 (6)	0.0216 (6)	0.0182 (6)	−0.0028 (5)	0.0016 (5)	−0.0011 (5)
O1	0.0283 (5)	0.0338 (6)	0.0224 (5)	0.0082 (5)	0.0086 (4)	0.0067 (4)
O2	0.0272 (5)	0.0203 (5)	0.0220 (5)	−0.0007 (4)	0.0058 (4)	0.0011 (4)
O3	0.0254 (5)	0.0293 (6)	0.0332 (6)	0.0024 (5)	0.0028 (4)	−0.0082 (5)
O4	0.0253 (5)	0.0249 (5)	0.0288 (5)	0.0079 (4)	0.0052 (4)	0.0010 (4)
O5	0.0210 (5)	0.0265 (5)	0.0280 (5)	−0.0002 (4)	0.0060 (4)	−0.0053 (4)
O6	0.0266 (5)	0.0192 (5)	0.0239 (5)	0.0018 (4)	0.0068 (4)	0.0018 (4)
O7	0.0207 (5)	0.0234 (5)	0.0226 (5)	0.0002 (4)	0.0049 (4)	0.0023 (4)
O8	0.0253 (5)	0.0230 (5)	0.0239 (5)	−0.0070 (4)	0.0048 (4)	−0.0011 (4)

### Geometric parameters (Å, º)

**Table:**

C1—N1	1.4776 (17)	C14—N2	1.4763 (17)
C1—C5	1.528 (2)	C14—C18	1.525 (2)
C1—C2	1.530 (2)	C14—C15	1.5263 (19)
C1—H1	0.98	C14—H14	0.98
C2—C3	1.530 (2)	C15—C16	1.524 (2)
C2—H2A	0.97	C15—H15A	0.97
C2—H2B	0.97	C15—H15B	0.97
C3—O2	1.4710 (17)	C16—O6	1.4775 (16)
C3—C4	1.521 (2)	C16—C17	1.525 (2)
C3—H3	0.98	C16—H16	0.98
C4—N1	1.4746 (18)	C17—N2	1.4742 (18)
C4—H4A	0.97	C17—H17A	0.97
C4—H4B	0.97	C17—H17B	0.97
C5—O1	1.1973 (18)	C18—O5	1.2053 (17)
C5—O2	1.3606 (17)	C18—O6	1.3576 (17)
C6—O3	1.2113 (18)	C19—O7	1.2158 (17)
C6—N1	1.3519 (19)	C19—N2	1.3423 (18)
C6—O4	1.3526 (17)	C19—O8	1.3475 (16)
C7—O4	1.4592 (18)	C20—O8	1.4648 (16)
C7—C8	1.497 (2)	C20—C21	1.494 (2)
C7—H7A	0.97	C20—H20A	0.97
C7—H7B	0.97	C20—H20B	0.97
C8—C9	1.387 (2)	C21—C26	1.388 (2)
C8—C13	1.396 (2)	C21—C22	1.392 (2)
C9—C10	1.379 (2)	C22—C23	1.388 (2)
C9—H9	0.93	C22—H22	0.93
C10—C11	1.393 (2)	C23—C24	1.380 (2)
C10—H10	0.93	C23—H23	0.93
C11—C12	1.380 (2)	C24—C25	1.393 (2)
C11—H11	0.93	C24—H24	0.93
C12—C13	1.385 (2)	C25—C26	1.383 (2)
C12—H12	0.93	C25—H25	0.93
C13—H13	0.93	C26—H26	0.93
			
N1—C1—C5	103.08 (11)	C15—C14—H14	116.7
N1—C1—C2	100.58 (11)	C16—C15—C14	91.73 (10)
C5—C1—C2	100.05 (11)	C16—C15—H15A	113.3
N1—C1—H1	116.8	C14—C15—H15A	113.3
C5—C1—H1	116.8	C16—C15—H15B	113.3
C2—C1—H1	116.8	C14—C15—H15B	113.3
C3—C2—C1	91.69 (11)	H15A—C15—H15B	110.7
C3—C2—H2A	113.3	O6—C16—C15	101.81 (10)
C1—C2—H2A	113.3	O6—C16—C17	105.66 (10)
C3—C2—H2B	113.3	C15—C16—C17	103.63 (11)
C1—C2—H2B	113.3	O6—C16—H16	114.8
H2A—C2—H2B	110.7	C15—C16—H16	114.8
O2—C3—C4	106.41 (11)	C17—C16—H16	114.8
O2—C3—C2	101.74 (11)	N2—C17—C16	99.60 (11)
C4—C3—C2	103.34 (12)	N2—C17—H17A	111.9
O2—C3—H3	114.6	C16—C17—H17A	111.9
C4—C3—H3	114.6	N2—C17—H17B	111.9
C2—C3—H3	114.6	C16—C17—H17B	111.9
N1—C4—C3	99.40 (11)	H17A—C17—H17B	109.6
N1—C4—H4A	111.9	O5—C18—O6	122.20 (13)
C3—C4—H4A	111.9	O5—C18—C14	131.54 (13)
N1—C4—H4B	111.9	O6—C18—C14	106.07 (11)
C3—C4—H4B	111.9	O7—C19—N2	125.19 (13)
H4A—C4—H4B	109.6	O7—C19—O8	124.89 (12)
O1—C5—O2	122.78 (14)	N2—C19—O8	109.92 (12)
O1—C5—C1	131.28 (13)	O8—C20—C21	105.41 (11)
O2—C5—C1	105.77 (11)	O8—C20—H20A	110.7
O3—C6—N1	124.85 (13)	C21—C20—H20A	110.7
O3—C6—O4	125.12 (13)	O8—C20—H20B	110.7
N1—C6—O4	109.97 (12)	C21—C20—H20B	110.7
O4—C7—C8	107.58 (12)	H20A—C20—H20B	108.8
O4—C7—H7A	110.2	C26—C21—C22	119.05 (13)
C8—C7—H7A	110.2	C26—C21—C20	119.26 (13)
O4—C7—H7B	110.2	C22—C21—C20	121.62 (13)
C8—C7—H7B	110.2	C23—C22—C21	120.20 (14)
H7A—C7—H7B	108.5	C23—C22—H22	119.9
C9—C8—C13	118.47 (14)	C21—C22—H22	119.9
C9—C8—C7	120.22 (14)	C24—C23—C22	120.46 (14)
C13—C8—C7	121.29 (14)	C24—C23—H23	119.8
C10—C9—C8	121.21 (14)	C22—C23—H23	119.8
C10—C9—H9	119.4	C23—C24—C25	119.60 (14)
C8—C9—H9	119.4	C23—C24—H24	120.2
C9—C10—C11	119.95 (15)	C25—C24—H24	120.2
C9—C10—H10	120.0	C26—C25—C24	119.89 (15)
C11—C10—H10	120.0	C26—C25—H25	120.1
C12—C11—C10	119.38 (15)	C24—C25—H25	120.1
C12—C11—H11	120.3	C25—C26—C21	120.76 (13)
C10—C11—H11	120.3	C25—C26—H26	119.6
C11—C12—C13	120.54 (14)	C21—C26—H26	119.6
C11—C12—H12	119.7	C6—N1—C4	127.24 (12)
C13—C12—H12	119.7	C6—N1—C1	123.02 (11)
C12—C13—C8	120.43 (14)	C4—N1—C1	108.53 (11)
C12—C13—H13	119.8	C19—N2—C17	127.75 (12)
C8—C13—H13	119.8	C19—N2—C14	123.84 (11)
N2—C14—C18	102.83 (10)	C17—N2—C14	108.07 (11)
N2—C14—C15	100.74 (11)	C5—O2—C3	106.52 (11)
C18—C14—C15	100.54 (11)	C6—O4—C7	115.82 (11)
N2—C14—H14	116.7	C18—O6—C16	106.04 (10)
C18—C14—H14	116.7	C19—O8—C20	115.16 (10)

### Hydrogen-bond geometry (Å, º)

**Table:**

*D*—H···*A*	*D*—H	H···*A*	*D*···*A*	*D*—H···*A*
C1—H1···O5^i^	0.98	2.42	3.3493 (18)	159
C2—H2*A*···O7^ii^	0.97	2.46	3.2116 (18)	134
C3—H3···O5^iii^	0.98	2.37	3.2816 (18)	155
C4—H4*B*···O7^iii^	0.97	2.39	3.3408 (18)	168
C15—H15*A*···O3^iv^	0.97	2.44	3.1382 (19)	128
C16—H16···O1^v^	0.98	2.49	3.2207 (18)	131
C26—H26···O6^vi^	0.93	2.58	3.4584 (19)	157

Symmetry codes: (i) *x*−1, *y*−1, *z*; (ii) *x*−1, *y*, *z*; (iii) −*x*+1, *y*−1/2, −*z*+1; (iv) *x*+1, *y*+1, *z*; (v) −*x*+1, *y*+1/2, −*z*; (vi) *x*, *y*−1, *z*.
