# Supplementary figures and images for: Crystal structure of benzyl 3-oxo-2-oxa-5-aza­bicyclo­[2.2.1]heptane-5-carboxyl­ate
Source: Acta Crystallogr E Crystallogr Commun. 2015 Jun 6;71(Pt 7):o447–8. doi: 10.1107/S2056989015010464 (PMC4518953; doi:10.1107/S2056989015010464)

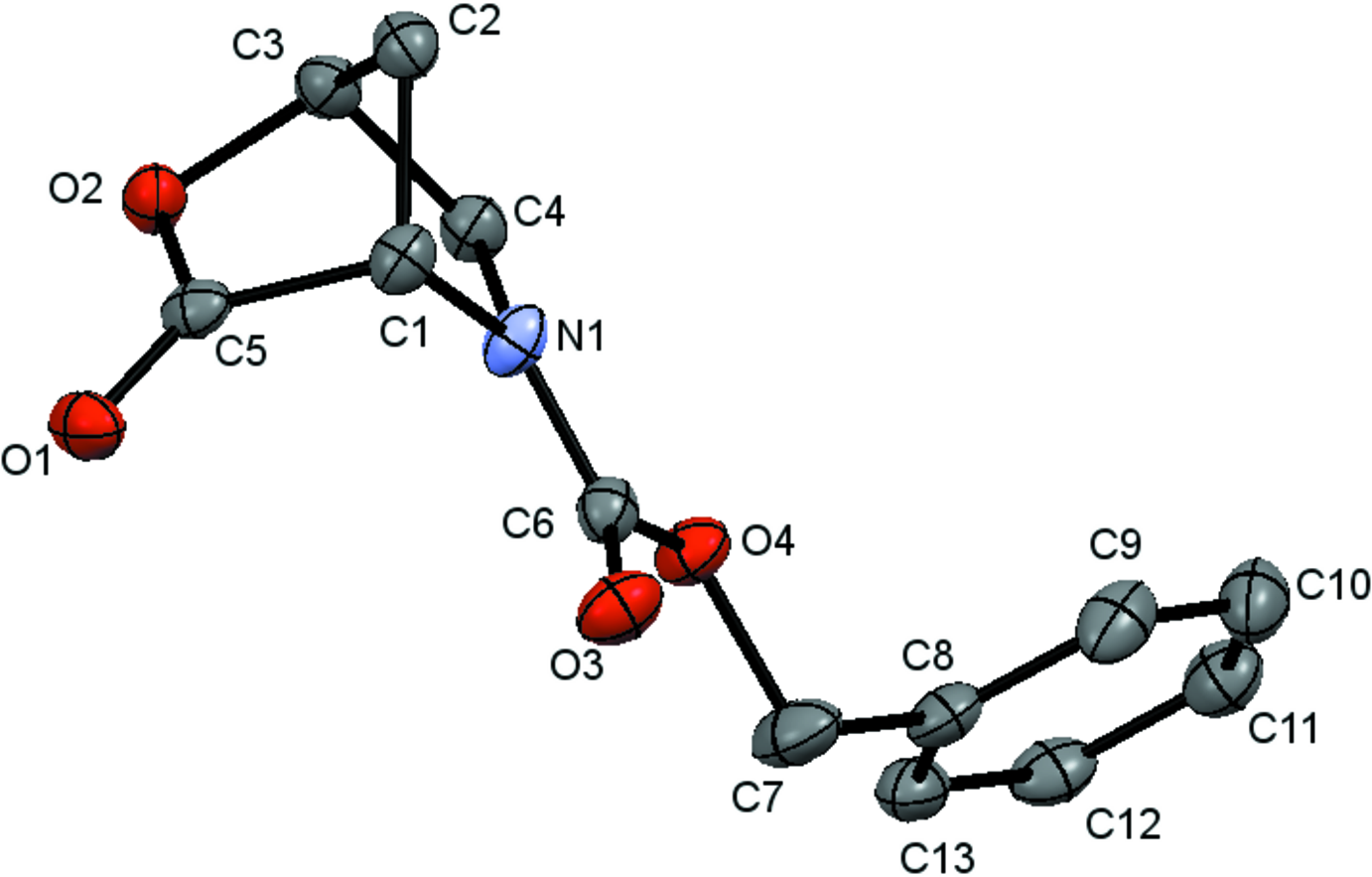

Supplement: Supplementary file 3 [file e-71-0o447-fig1.tif]

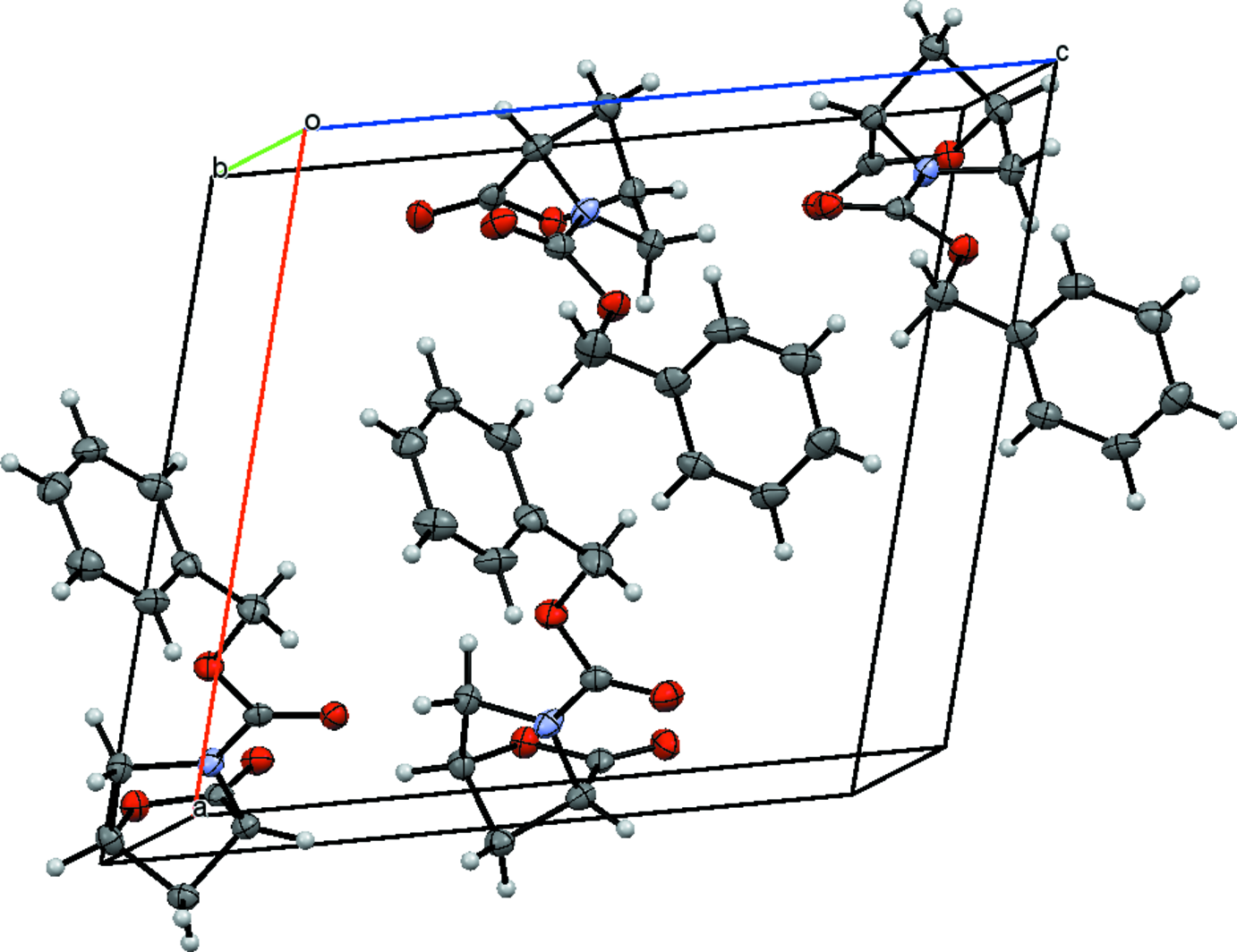

Supplement: Supplementary file 4 [file e-71-0o447-fig2.tif]
